# Proteomic signatures reflect effects of semaglutide treatment for MASH^☆^

**DOI:** 10.1016/j.jhepr.2025.101521

**Published:** 2025-07-22

**Authors:** Jörn M. Schattenberg, Henning Grønbæk, Iris Kliers, Steen Ladelund, Michelle T. Long, Sune Boris Nygård, Arun J. Sanyal, Melanie J. Davies

**Affiliations:** 1 Department of Internal Medicine II, Saarland University Medical Center, Homburg, Germany; 2 Department of Hepatology & Gastroenterology, Aarhus University Hospital, and Department of Clinical Medicine, Aarhus University, Aarhus, Denmark; 3 Novo Nordisk A/S, Søborg, Denmark; 4 Stravitz-Sanyal Institute for Liver Disease and Metabolic Health, School of Medicine, Virginia Commonwealth University School of Medicine, Richmond, VA, USA; 5 Diabetes Research Centre, University of Leicester, Leicester, UK; 6 NIHR Leicester Biomedical Research Centre, Leicester, UK

**Keywords:** Metabolic Dysfunction-Associated Steatohepatitis, Obesity, Type 2 Diabetes, Fibrosis, Inflammation, Ballooning

## Abstract

**Background & Aims:**

Proteomic technology has emerged as a non-invasive method for grading and staging metabolic dysfunction-associated steatohepatitis (MASH). The aims of this *post hoc* analysis of the STEP 1 and STEP 2 trials were to assess the distribution of SomaSignal-derived MASH components, evaluate the effect of semaglutide (2.4 mg and 1.0 mg) on these components, and test concordance with biopsy-based MASH histology from the phase IIb Sema-MASH trial.

**Methods:**

Participants with overweight or obesity (STEP 1) and type 2 diabetes (STEP 2), who received once-weekly semaglutide or placebo for 68 weeks and had available biological samples, were included. In addition, participants with biopsy-confirmed MASH from the phase IIb Sema-MASH trial who received once-daily semaglutide or placebo for 72 weeks were included.

**Results:**

SomaSignal-derived steatosis was prevalent in both STEP 1 (43.2%) and STEP 2 (71.7%). At week 68, participants who received semaglutide had significantly lower odds of exhibiting SomaSignal-defined MASH components compared with those on placebo. They were also more likely to have a less severe SomaSignal-derived stage of metabolic dysfunction-associated steatotic liver disease, with odds ratios for semaglutide 2.4 mg of 5.26 (95% CI 3.59–7.72) in STEP 1 and 4.90 (95% CI 2.86–8.40) in STEP 2; both *p* <0.0001. SomaSignal-derived MASH components correlated with histologic changes, standard liver-related biomarkers, and measures of glycemic control.

**Conclusions:**

These findings suggest that SomaSignal MASH component testing may be useful for evaluating the potential therapeutic effects of semaglutide in patients with MASH and related comorbidities. However, prospective randomized clinical trials are necessary to confirm these results.

**Clinical trial registration numbers:**

STEP 1: NCT03548935; STEP 2: NCT03552757; MASH phase IIb trial: NCT02970942.

**Impact and implications:**

Liver biopsy is the current gold standard for the diagnosis of metabolic dysfunction-associated steatohepatitis (MASH); however, this procedure is invasive and time consuming for patients living with MASH. The use of non-invasive tests to diagnose MASH is becoming increasingly popular and many clinicians see this approach as the diagnostic future. SomaSignal is a proteomics-based model designed to non-invasively test for and validate MASH components as well as detect the prevalence of metabolic dysfunction-associated steatotic liver disease stages, against biopsy results. In the future, SomaSignal MASH components may have an impact on future clinical practice by providing clinicians with an alternative option for non-invasive assessment of treatment effects in the early clinical stages of therapeutic development.

## Highlights

• SomaSignal-derived steatosis was highly prevalent in the STEP 1 and 2 trials.
• Participants receiving semaglutide had a less severe SomaSignal-derived MASLD stage.
• MASH components correlated with histologic change.

## Graphical abstract

**Figure:**
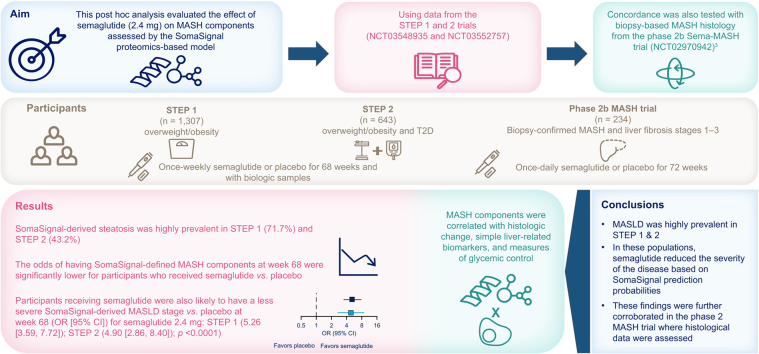

## Introduction

The global prevalence of metabolic dysfunction-associated steatotic liver disease (MASLD)^1^ among adults is around 30% and continues to rise,^2^^,^^3^ with associated high socioeconomic costs.^3^^,^^4^ Patients living with MASLD have cardiometabolic risk factors including obesity, insulin resistance, and type 2 diabetes (T2D).^5^ There remains a need for improved non-invasive assessment of MASLD and monitoring of treatment response in these patient populations. The disease spectrum of MASLD ranges from simple steatosis to metabolic dysfunction-associated steatohepatitis (MASH), fibrosis, and cirrhosis, with increasing severity of liver scarring ranging histologically from delicate pericellular fibrosis to complete distortion of normal architecture and formation of regeneration noduli in the cirrhotic stage.^3^ Fibrosis stage is prognostic and, accordingly, the presence of clinically significant fibrosis in MASH increases the risk of cirrhosis and hepatocellular carcinoma,^2^ along with liver-related morbidity and mortality.^6^ It is also associated with impaired quality of life.^7^

The current gold standard for diagnosing MASH is liver biopsy, which is time consuming, invasive, and associated with complications.^8^^,^^9^ In addition, sampling error can occur due to heterogeneity in the liver, resulting in disease severity being inaccurately characterized.^10^ Lifestyle modifications (*e.g*. diet, exercise, and weight loss) are the primary treatment of MASH; however, these are not always effective at achieving and retaining positive results.^6^^,^^11^ Recently, resmetirom has been approved by the US Food and Drug Administration for the treatment of noncirrhotic MASH with moderate to advanced liver fibrosis, consistent with fibrosis stages F2 and F3;^12^^,^^13^ however, there remains a need for additional treatment options.^14^^,^^15^

Semaglutide is a glucagon-like peptide-1 receptor agonist approved for the treatment of T2D, and to reduce the risk of major cardiovascular events in people with T2D and cardiovascular disease.^16^^,^^17^ Semaglutide is also approved for the treatment of overweight/obesity and to reduce the risk of major cardiovascular events in people with cardiovascular disease and obesity/overweight.^18^ Semaglutide has demonstrated clinical benefit in metabolic dysfunction-associated steatohepatitis (MASH).^19^ In the ongoing phase III ESSENCE trial (NCT04822181) involving participants with biopsy-defined MASH and fibrosis stage 2 or 3, an interim analysis of the first 800 randomized participants showed that once-weekly subcutaneous (s.c.) semaglutide 2.4 mg was superior to placebo for improvement in liver fibrosis as well as resolution of steatohepatitis after 72 weeks of treatment.^20^

Given the limitations of liver biopsy, non-invasive tests are required to reliably assess MASH components and monitor treatment effects in populations with obesity, both with and without T2D. Recently, a targeted, non-invasive proteomics model,^21^ derived from patients with histologically defined MASLD, successfully identified diagnostic signatures of the key histologic components of MASH (*e.g.* steatosis, hepatocyte ballooning, lobular inflammation, and fibrosis), predicted patients with at-risk MASH, and detected histologic changes in MASH for three different drugs.^22^^,^^23^

In the current *post hoc* analysis, we aimed to determine the distribution of MASH components, and the impact of semaglutide treatment on these MASH components, using a proteomic panel based on measurement of aptamers in serum (SomaLogic, Boulder, CO, USA) among participants with related comorbidities. To achieve this, we assessed the distribution of SomaSignal-derived MASH components and MASLD in a population with obesity, with or without T2D, evaluating the treatment effects of semaglutide using data from the STEP 1 and STEP 2 trials.^24^^,^^25^ We further corroborated our findings by evaluating concordance in changes in SomaSignal-derived MASH components with biopsy-based data from the phase IIb MASH trial and related these to changes in histology.^19^ Using the STEP 1 and 2 data, we also investigated prediction probabilities and concordance of SomaSignal-derived MASH components with simple liver-related biomarkers. Moreover, we investigated the prediction probabilities and concordance of SomaSignal-derived MASLD components (steatosis) with measures of glycemic control including glycated hemoglobin (HbA1c) and homeostatic model assessment of insulin resistance (HOMA-IR).

## Materials and methods

This is a *post hoc* analysis of three previously published randomized trials.^19^^,^^24^^,^^25^ The protocol and amendments for each trial were approved by the relevant institutional review board or independent ethics committee at each study site.^19^^,^^24^^,^^25^ Briefly, STEP 1 (NCT03548935) was a double-blind, placebo-controlled phase IIIa trial involving adults with overweight/obesity (BMI ≥30 kg/m^2^ or ≥27 kg/m^2^ in persons with ≥1 weight-related comorbidity). Participants were randomized 2:1 to receive once-weekly s.c. semaglutide 2.4 mg or placebo for 68 weeks including 16 weeks of dose-escalation. STEP 2 (NCT03552757) was a double-blind, placebo-controlled phase IIIa trial involving adults with overweight/obesity (BMI ≥27 kg/m^2^) and T2D (HbA_1c_ 7–10%). Participants were randomized 1:1:1 to receive either once-weekly s.c. semaglutide 1.0 mg, semaglutide 2.4 mg, or placebo for 68 weeks, including 16 weeks of dose-escalation. In STEP 1 and STEP 2, human biological samples for future research were collected at week 0 and week 68 from all participants who provided consent. Some countries had reduced or no SomaSignal data due to a lower proportion of people consenting to sample collection or a lack of local ethical approval for data collection/processing.

The phase IIb trial (NCT02970942) included in the current analysis was a double-blind, placebo-controlled, parallel-group, dose-finding trial involving participants with biopsy-confirmed MASH, a nonalcoholic fatty liver disease activity score of 4 or more with a subscore of ≥1 for each MASH component (steatosis, hepatocyte ballooning, and lobular inflammation), and liver fibrosis stages F1–F3 (each assessed by central pathologist evaluation based on MASH Clinical Research Network criteria),^26^ with or without T2D and a BMI of >25 kg/m^2^ at screening. Participants were randomized to receive either once-daily s.c. semaglutide 0.1 mg, 0.2 mg, 0.4 mg (corresponding to 2.4 mg once weekly), or placebo for 72 weeks, including a 4- to 16-week dose-escalation period.^19^ Liver biopsies were performed at week 0 and week 72. Participants had serum collected at baseline, weeks 28, 52, and 72. Only baseline and week-72 data are used in the current analysis.

### SomaSignal prediction probabilities

SomaSignal MASH tests are modified, aptamer-based, elastic net logistic regression models trained and validated against biopsy results for each MASH component (*i.e*. steatosis [containing 12 protein analytes], lobular inflammation [containing 14 protein analytes], hepatocyte ballooning [containing five protein analytes], and fibrosis [containing eight protein analytes]).^22^ The test is currently available for research use only.

In the current analysis, prediction probabilities for SomaSignal-derived MASH components at baseline and at the end of the trial were provided by SomaLogic using the SomaSignal models, with higher probabilities indicating a higher likelihood of more advanced liver pathology. Dichotomized SomaSignal MASH components derived in the current analysis were steatosis (grade 1 to 3 *vs.* 0; linked to clearance of steatosis), lobular inflammation (grade 2 to 3 *vs.* 0 to 1), hepatocyte ballooning (grade 1 to 2 *vs.* 0), and fibrosis (stage 0 to 1 *vs.* 2 to 4). The SomaSignal scores provide a probability of whether fibrosis is greater than or equal to stage 2 (at least clinically significant fibrosis). The SomaSignal MASH components were originally derived in this manner because of linkage to criteria for MASH resolution and identification of clinically significant fibrosis – please see Sanyal *et al.*^22^ for further details.

### Mapping to MASLD staging

SomaSignal-derived MASH components were also used to determine MASLD stage. Staging was defined as non-MASLD (severity = 0; reference stage) with no MASH components present. Presence of MASLD was defined as: simple steatosis (severity = 1), with only steatosis present and no other MASH components; indeterminate (severity = 2), with some, but not all, MASH components (any participant that had combinations of steatosis, lobular inflammation, or hepatocyte ballooning, or at least clinically significant fibrosis [stage F2 or higher], but not steatosis alone and not meeting the MASH definition); or MASH (severity = 3) with steatosis and lobular inflammation present, and with hepatocyte ballooning in sufficient amounts to no longer meet MASH-resolution criteria irrespective of SomaSignal fibrosis category. MASH (severity = 3) was further divided according to the fibrosis category, *i.e.* with or without at least clinically significant fibrosis (stage F2 or higher).

### Outcomes and analysis

The efficacy of semaglutide (2.4 mg STEP 1 and STEP 2, 1.0 mg STEP 2 only) *vs.* placebo was analyzed for the STEP 1 and 2 trials by presence or absence of MASH components, using a binary classifier derived from the SomaSignal prediction probabilities (prediction probability threshold of ≥0.5) at the end of the trial, using mixed effects logistic regression for repeated measurements. Odds ratios at the end of the trial were calculated from continuous prediction probabilities using analysis of covariance on the log-odds scale. Odds ratios of having a less severe MASLD stage at the end of the trial used mixed effects proportional odds regression for repeated measurements.

The association of changes in prediction probabilities for the SomaSignal-derived MASH components and improvement in equivalent histologic endpoints from biopsy was assessed using odds ratios at the end of the trial, applying logistic regression adjusted for treatment group and baseline prediction probability value.

Associations between SomaSignal-derived MASH components and simple liver-related biomarkers and measures of glycemic control were also assessed. Prediction probabilities for clinically significant fibrosis, based on the Fibrosis-4 (FIB-4) index in STEP 1 and STEP 2, were analyzed using linearized prediction probabilities via logit transformation. FIB-4 score categories were defined as 0–<1.3 (low), ≥1.3–<2.67 (intermediate), and ≥2.67 (high). An age-dependent FIB-4 threshold (age ≥65 years: 2–2.67) was also used to improve specificity.^27^ Pearson’s correlation coefficients were calculated between prediction probabilities for SomaSignal-derived MASH components and FIB-4 score, alanine aminotransferase (ALT), aspartate aminotransferase (AST), and platelets, and between steatosis prediction probabilities for HbA1c and HOMA-IR. All correlation scatter plots were enriched with a kernel smoother (loess).

Data from STEP 1 and 2 were analyzed by SomaLogic using the 7k version of the SomaScan assay. Data from the semaglutide phase IIb MASH trial were analyzed using the 5k version. All three trials were analyzed from serum samples on the SomaScan 4.1 platform. SomaLogic translated the MASH SomaSignal predictors between the 5k and 7k versions. All analyzed data are observed data, for the on-treatment period.

## Results

### Participants

In STEP 1 and 2, 1,307 of 1,961 (66.6%) participants and 643 of 1,210 (53.1%) participants, respectively, had available SomaSignal data and were included (Table 1). Most participants were White (75.1% and 60.0%, respectively) with a mean age of 47.5 years and 56.3 years, respectively. In STEP 1, almost three-quarters of participants were women (72.8%) while in STEP 2, approximately half were women (49.9%) (Table 1).

**Table 1:** Baseline characteristics for participants in STEP 1 and 2 and the phase IIb MASH trial.

	STEP 1 n = 1,307	STEP 2 n = 643	Phase IIb trial n = 234
Age, years			
Mean (SD)	47.5 (12.7)	56.3 (10.8)	55.1 (10.7)
18 to <65	1,190 (91.0)	487 (75.7)	192 (82.1)
≥65	117 (9.0)	156 (24.3)	42 (17.9)
Sex			
Women	951 (72.8)	321 (49.9)	138 (59.0)
BMI, kg/m^2^			
<30	79 (6.0)	122 (19.0)	56 (23.9)
≥30 to <35	431 (33.0)	231 (35.9)	64 (27.4)
≥35 to <40	401 (30.7)	147 (22.9)	57 (24.4)
≥40	396 (30.3)	143 (22.2)	57 (24.4)
Type 2 diabetes	0 (0.0)	643 (100.0)	149 (63.7)
eGFR, ml/min/1.73m^2^			
Normal (≥90)	856 (65.5)	416 (64.7)	165 (71.4)
Mild RI (≥60 to <90)	431 (33.0)	190 (29.5)	59 (25.5)
Moderate RI (≥30 to <60)	20 (1.5)	37 (5.8)	7 (3.0)
Race			
White	981 (75.1)	386 (60.0)	173 (73.9)
Asian	161 (12.3)	183 (28.5)	41 (17.5)
Black or African American	65 (5.0)	55 (8.6)	1 (0.4)
Other∗	100 (7.7)	19 (3.0)	19 (8.1)

Data are n (%) unless otherwise stated.

eGFR, estimated glomerular filtration rate; MASH, metabolic dysfunction-associated steatohepatitis; RI, renal impairment.

∗ Includes American Indian or Alaska Native, Native Hawaiian or other Pacific Islander, other, and not reported.

Of the 320 participants included in the phase IIb MASH trial, 234 (73.1%) had available data. Most participants were White (73.9%) with a mean age of 55.1 years, and approximately 60% were women (Table 1).

Baseline characteristics for the full analysis set from the primary trials are presented in Table S1; there were no clinically meaningful differences between the subgroup of participants included in these analyses and the full trial populations.

### Distribution of SomaSignal-derived MASH components and MASLD stages in STEP 1 and STEP 2

In STEP 1, when SomaSignal MASH components were assessed independently, 43.2% of participants had steatosis at baseline, while 5.0%, 3.8%, and 2.4% had lobular inflammation, hepatocyte ballooning, and at least clinically significant fibrosis (stage F2 or higher), respectively (Table 2). In STEP 2, a larger proportion of participants had steatosis at baseline (71.7%) while 25.0%, 21.5%, and 21.9% had lobular inflammation, hepatocyte ballooning, and at least clinically significant fibrosis (stage F2 or higher), respectively (Table 2).

**Table 2:** Baseline distribution of SomaSignal-derived MASH components and MASLD staging in STEP 1 and 2.

	STEP 1 n = 1,307	STEP 2 n = 643
**MASH components, %**		
Steatosis (grade 1 to 3)	43.2	71.7
Lobular inflammation (grade 2 to 3)	5.0	25.0
Hepatocyte ballooning (grade 1 to 2)	3.8	21.5
Significant fibrosis (stage 2 to 4)	2.4	21.9

**MASLD stage, %**		
Non-MASLD	55.5	25.2
Simple steatosis	36.0	38.7
Indeterminate	7.2	21.0
MASH	1.4	15.1
MASH with at least clinically significant fibrosis (stage 2 to 4)	0.4	11.5

Data are not histologic; MASH components and MASLD stages are derived from SomaSignal data. MASLD, metabolic dysfunction-associated steatotic liver disease; MASH, metabolic dysfunction-associated steatohepatitis.

In STEP 1, according to MASLD staging, 36.0% of participants had simple steatosis (MASLD), 1.4% had MASH, and 0.4% of participants had MASH with at least clinically significant fibrosis (stage F2 or higher) (Table 2). Correspondingly, in STEP 2, 38.7% of participants had simple steatosis (MASLD), 15.1% had MASH, and 11.5% of participants had MASH with at least clinically significant fibrosis (stage F2 or higher) (Table 2).

### Semaglutide treatment effect on SomaSignal-derived MASH components and MASLD stage

**Fig. 1:**
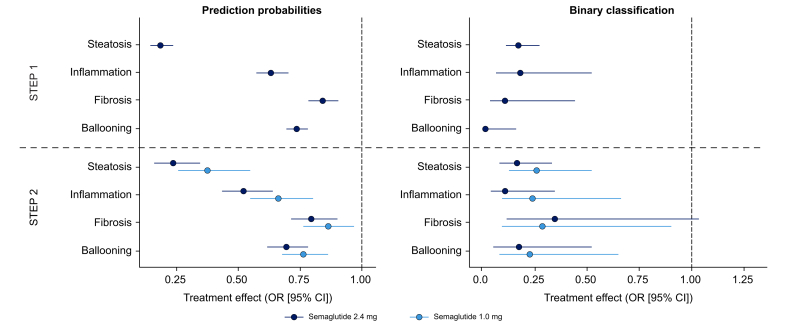
ORs for the presence of MASH components in STEP 1 and 2. Prediction probability based on analysis of covariance on the log-odds scale. Binary classifier based on repeated measurement mixed logistic regression. Estimate is on OR scale (*i.e*. 1 is equivalent to no treatment effect). Criteria used to define fibrosis was stage 0 to 1 *vs.* 2 or higher (clinically significant fibrosis). MASH, metabolic dysfunction-associated steatohepatitis; OR, odds ratio.

For both continuous prediction probabilities and binary classification (where participants had a theoretical biopsy result), the odds of having SomaSignal-defined MASH components at the end of the trial were significantly lower for participants who received semaglutide *vs.* placebo (Fig. 1). For continuous prediction probabilities, the treatment effects of semaglutide 2.4 mg were statistically significant across all SomaSignal-derived MASH components in STEP 1. The effect was most pronounced for steatosis, followed by effects on lobular inflammation, hepatocyte ballooning, and fibrosis. In STEP 2, similar effect sizes were observed for semaglutide 2.4 mg, and the effect appeared to be dose dependent across all SomaSignal-derived MASH components when comparing 1.0 mg and 2.4 mg dose levels (Fig. 1). For the binary classification analysis, statistically significant semaglutide treatment effects were observed across all SomaSignal-derived MASH components in STEP 1 and STEP 2 (Fig. 1).

The odds ratios for participants having a less severe stage of MASLD at week 68 (based on having greater reduction or less progression of MASLD) were significantly greater for participants receiving semaglutide 2.4 mg (STEP 1 and STEP 2) and 1.0 mg (STEP 2 only) *vs.* placebo (all comparisons *p* <0.0001) (Table 3).

**Table 3:** Odds ratios for less severe MASLD stage at the end of the trial for semaglutide *vs.* placebo in STEP 1 and 2.

	Odds ratio (95% CI)	*p* value
STEP 1 semaglutide 2.4 mg	5.26 (3.59–7.72)	<0.0001
STEP 2 semaglutide 2.4 mg	4.90 (2.86–8.40)	<0.0001
STEP 2 semaglutide 1.0 mg	3.62 (2.13–6.15)	<0.0001

Less severe MASLD was defined as having greater reduction or less progression of MASLD. Based on a mixed model for logistic regression.

MASLD, metabolic dysfunction-associated steatotic liver disease.

### Concordance of SomaSignal-derived MASH components with histology

**Fig. 2:**
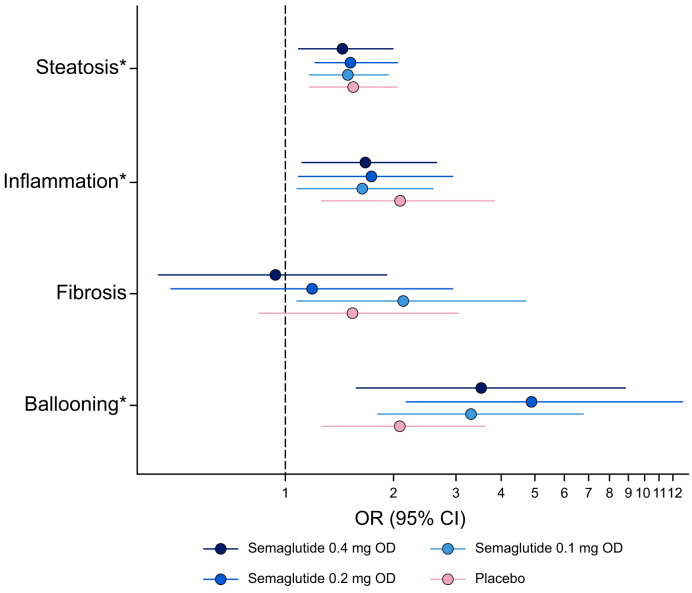
ORs for improvement in histology from baseline to the end of the trial as SomaSignal prediction probabilities decrease in the phase IIb MASH trial. Based on logistic regression. OR is for a decrease of one unit in prediction probability on the logit scale. Outcome is ≥1 stage improvement in histology *vs.* stable or progression. ∗ORs for the treatment groups are statistically significantly different. MASH, metabolic dysfunction-associated steatohepatitis; OD, once daily; OR, odds ratio. Fig. adapted from: Newsome PN *et al. N Eng J Med* 2021;384:1113–24.

**Fig. 3:**
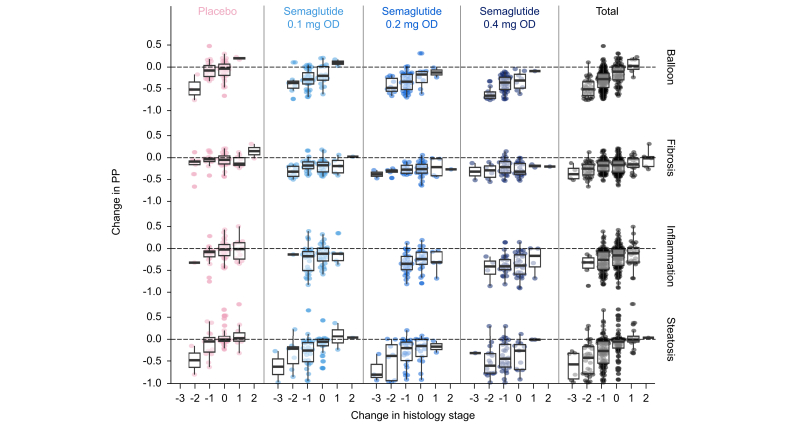
Change from baseline in SomaSignal prediction probabilities and change in histology at 72 weeks in the phase IIb MASH trial. Total column includes semaglutide and placebo. Fibrosis was defined as stage 0 to 1 *vs.* 2 to 4. The SomaSignal scores provide a probability of whether fibrosis is greater than or equal to stage 2 (at least clinically significant fibrosis). MASH, metabolic dysfunction-associated steatohepatitis; OD, once daily; PP, prediction probability.

A decrease in prediction probability for the SomaSignal-derived MASH components of steatosis, lobular inflammation, and hepatocyte ballooning was associated with an improvement in histology regardless of semaglutide treatment group in the phase IIb MASH trial (Fig. 2, Fig. 3). For clinically significant fibrosis (stage F2 or higher), a decrease in the prediction probability was not statistically associated with a ≥1 stage improvement in histology *vs.* stable disease or progression (Fig. 2). The change from baseline in SomaSignal prediction probability for clinically significant fibrosis and change in histology at 72 weeks showed a non-significant trend (Fig. 3).

### Prediction probabilities and concordance of SomaSignal-derived MASH components with liver-related biomarkers

**Fig. 4:**
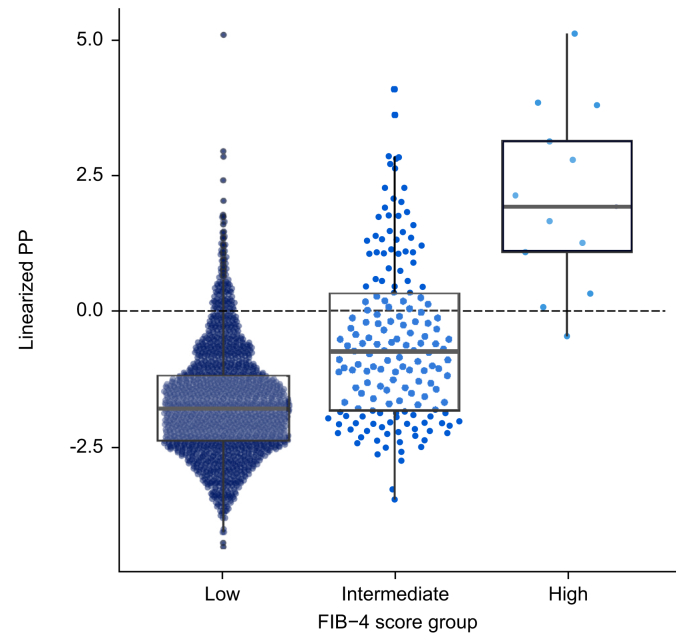
Linearized PP by FIB-4 score in STEP 1 and 2. Linearized PP: logit-transformed PP. The dashed line represents the threshold for the binary classifier. FIB-4, Fibrosis-4 index; PP, prediction probability.

Across STEP 1 and 2, SomaSignal-derived clinically significant MASH fibrosis (stage F2 or higher) correlated with FIB-4 score (Fig. 4). In STEP 2, in which participants had a higher burden of SomaSignal-derived clinically significant fibrosis at baseline, a larger correlation was observed between FIB-4 score and clinically significant fibrosis than in STEP 1 (0.27 [95% CI 0.23–0.31] *vs.* 0.12 [95% CI 0.09–0.14], respectively) (Fig. S1).

The association was maintained for AST, ALT, and thrombocytes at baseline, where the correlations were generally greater in participants with T2D in STEP 2 than those without T2D in STEP 1 (Fig. S2). There were also correlations between SomaSignal prediction probabilities for MASH ballooning and AST and ALT at baseline that were greater in participants with T2D (STEP 2) than in those without (STEP 1) (0.41 [95% CI 0.38–0.44] and 0.37 [95% CI 0.33–0.40] *vs.* 0.20 [95% CI 0.18–0.23] and 0.20 [95% CI 0.17–0.22], respectively) (Fig. S3). Correlations were observed between SomaSignal prediction probabilities for MASH inflammation and AST and ALT at baseline, with greater correlations in STEP 2 than STEP 1 (0.37 [95% CI 0.34–0.41] and 0.41 [95% CI 0.38–0.45] *vs.* 0.20 [95% CI 0.17–0.22] and 0.23 [95% CI 0.20–0.26]) (Fig. S4). The correlations between SomaSignal prediction probabilities for MASH steatosis and AST and ALT were generally similar in STEP 1 *vs.* STEP 2 (0.22 [95% CI 0.20–0.25] and 0.31 [95% CI 0.28–0.33] *vs.* 0.26 [95% CI 0.22–0.30] and 0.28 [95% CI 0.24–0.31], respectively) (Fig. S5).

### Prediction probabilities and concordance of SomaSignal-derived MASLD components with metabolic risk factors

In both STEP 1 and 2, a correlation was observed between prediction probabilities for SomaSignal-derived steatosis and HbA1c at baseline (Fig. S6). A stronger association was observed in STEP 1 *vs.* STEP 2 (0.19 [95% CI 0.13–0.24] *vs.* 0.10 [95% CI 0.02–0.17], respectively) (Fig. S6). For HOMA-IR and prediction probabilities for SomaSignal-derived steatosis, a positive correlation was observed that was comparable between both STEP 1 and 2 at baseline (0.37 [95% CI 0.32–0.42] and 0.34 [95% CI 0.27–0.40], respectively) (Fig. S7).

## Discussion

Non-invasive diagnostic tests for MASLD and MASH are urgently needed. Based on two pharmacological weight-loss trials and one liver-histology-based MASH study, we applied SomaSignal proteomics to describe the prevalence of SomaSignal-defined disease stages. In this *post hoc* analysis, we first assessed the distribution of SomaSignal-derived MASH components and MASLD staging in participants with obesity with and without T2D in two trials of semaglutide. Steatosis was highly prevalent in participants with overweight/obesity at baseline, and MASH with or without fibrosis was present in approximately 15% of participants with overweight/obesity and T2D in STEP 2. This rate can be considered relatively low *vs.* the global prevalence of MASH in people with overweight/obesity and reports from the literature.^2^^,^^3^ In a systematic review and meta-analysis, MASH without fibrosis was observed in approximately 34% of those with overweight/obesity.^28^ However, approximately 21% of participants with overweight and 12% of participants with obesity had fibrosis stage F2 and F3, respectively,^28^ which is comparable to the current analysis. Furthermore, in another systematic review and meta-analysis, the global prevalence of MASH among individuals with T2D was approximately 37%, with the prevalence of advanced fibrosis in those with T2D estimated at 17%.^29^ Across these studies, the prevalence of MASH with or without fibrosis was determined by liver biopsy.^28^^,^^29^

Differences in prevalence between the current analysis and existing literature are likely attributable to the methodological differences arising from the specific study populations (exclusion criteria) and the use of the SomaSignal test. Specifically, the test is not sensitive in distinguishing between absence *vs.* low grades of hepatic inflammation, and therefore some participants could have been misclassified. Moreover, the dichotomized SomaSignal categories resulted in the presence of fibrosis being defined as ‘at least clinically significant fibrosis’ (stage F2 or higher), in comparison with the Quek *et al.* study that used histologic evidence of any stage of fibrosis (F1–F4).^28^ Furthermore, a greater proportion of participants had SomaSignal-derived MASH components at baseline in STEP 2 than STEP 1, which would be expected due to participants in STEP 2 being older than in STEP 1 and having T2D at baseline. Indeed, severity of disease (fibrosis in particular) has been shown to be more pronounced in older people and those with T2D.[29], [30], [31], [32] In addition, MASH diagnosis in the current study relied on all three activity components being present. However, suboptimal sensitivity for each MASH component may have led to low sensitivity for MASH diagnosis overall.

Next, we assessed the treatment effect of semaglutide on SomaSignal-derived MASH components. Overall, semaglutide had significant effects on the SomaSignal-derived MASH components in both trials. In the prediction probability-based analysis, the greatest treatment effect for semaglutide was observed for SomaSignal-derived MASH steatosis followed by lobular inflammation and hepatocyte ballooning, with the least effect seen for fibrosis across both STEP trials. This effect also appeared to be dose dependent in STEP 2, where two semaglutide doses (1.0 mg and 2.4 mg) were assessed. Furthermore, 68 weeks of semaglutide treatment was associated with a significantly less severe MASH stage at the end of the trial compared to placebo, based on greater reduction or less progression of disease. The treatment effect also appeared to be dose dependent, with a greater effect observed with semaglutide 2.4 mg *vs.* 1.0 mg in STEP 2. Broadly, these results support the benefits of semaglutide as a potential treatment option for patients living with MASLD/MASH. This finding has been corroborated by results from the ESSENCE trial.^20^

The final area of investigation in this *post hoc* analysis was to provide supportive evidence for the utility of SomaSignal-derived data by assessing the concordance of SomaSignal-derived MASH components with equivalent biopsy-based MASH histology, simple liver-related biomarkers, and measures of glycemic control using data from the phase IIb Sema-MASH trial.^19^ Here, change in SomaSignal prediction probability was significantly associated with histologic change for steatosis, lobular inflammation, and hepatocyte ballooning.^19^ These results suggest that the blood-based, SomaSignal-derived MASH components may accurately detect histologic changes in disease activity in patients living with biopsy-confirmed MASH. Comparable data from previous trials of potential pharmacologic treatments for MASH and from the current analysis provide supportive evidence that SomaSignal tests can be used to non-invasively track improvements in liver fibrosis and other histologic features. Previously, SomaSignal successfully tracked histologic improvement in steatosis, lobular inflammation, and hepatocyte ballooning across three drugs with different mechanisms of action.^22^ 10.13039/100014337Furthermore, in comparison with ADAPT, MACK-3, and PRO-C3, SomaSignal has also been shown to perform optimally as a screening tool for MASH with clinically significant fibrosis in the LITMUS project.^33^ In that study, the sensitivity and specificity of the SomaSignal test was reported as 0.67 and 0.82, respectively, for patients living with MASH and clinically significant fibrosis.^33^ In a study of pegbelfermin, a PEGylated human fibroblast growth factor 21 analog, the SomaSignal MASH component tests supported and exceeded the improvements detected by liver biopsy and showed a significant dose-response relationship with pegbelfermin.^34^

The FIB-4 score is a widely used laboratory metric, recommended by international guidelines as a non-invasive test for risk stratification in individuals with suspected fibrotic MASH.^1^^,^[35], [36], [37], [38] In the current study, the utility of SomaSignal was further supported through the correlations observed between FIB-4 score and SomaSignal-derived MASH fibrosis. Given the greater disease burden in the T2D population, the correlation between FIB-4 and SomaSignal-derived fibrosis was greater in STEP 2 than STEP 1, as would be expected. SomaSignal-derived MASH activity components (*e.g*. lobular inflammation, hepatocyte ballooning, and steatosis) were also concordant with AST and ALT. Again, this was expected, given that these biomarkers are hepatocellular measures of liver inflammation and injury,^39^ and, similarly, this observation was most apparent in STEP 2. There are currently few other non-invasive tests available for the detection of MASH fibrosis. In a study by Chan *et al.*,^40^ the performance of non-invasive tests for diagnosing MASH fibrosis was compared, and SomaSignal detected a 46% prevalence of MASH fibrosis – outperforming most other non-invasive tests, including MACK-3, the NIS4 algorithm, the MAST score, and a proteomic-based classification model.^40^

Insulin resistance (assessed here by HOMA-IR) is a key metabolic factor underlying MASLD, and poor glycemic control, as measured by HbA1c, is acknowledged as a significant cardiometabolic risk factor for MASLD.^41^ In the current analysis, we were able to demonstrate an association between SomaSignal-derived MASH steatosis with both HOMA-IR and HbA1c. This finding supports evidence that the extent of endocrine dysregulation in MASH correlates with the presenting liver-related phenotype and progression of disease.^42^

There are several strengths and limitations to this analysis. Firstly, using data from STEP 1 and 2 involving participants with overweight/obesity with and without T2D allowed us to characterize MASH components in these specific populations. Furthermore, the STEP populations with associated comorbidities are generalizable to individuals in a real-world setting. As the ESSENCE trial results were not published at the time these analyses were conducted, using the SomaSignal test in the completed STEP studies allowed us to evaluate the distribution of MASH components in these relevant patient groups, and to explore any potential treatment effects of semaglutide on these components. Recently, two phase II trials (one investigating survodutide and one investigating tirzepatide) demonstrated a significant improvement in resolution of MASH with no worsening of fibrosis *vs.* placebo.^14^^,^^15^ Limitations included a lack of liver histologic data in the STEP program, therefore there is a possibility that some participants could have been misclassified in terms of fibrosis staging. In addition, the SomaSignal test is not sensitive for mild fibrosis, so it is possible that mild fibrosis was present in this population at a higher prevalence. Furthermore, it is not clear what the full clinical implications of changes in SomaSignal tests would be for the STEP 1 and 2 populations. However, we provide supportive evidence that changes in SomaSignal tests could be associated with histologic improvements, and this is a significant strength of the current study. While a trend was observed, the relationship between prediction probabilities for SomaSignal-derived MASH fibrosis and histology was not significant. However, histologic fibrosis improvement occurred infrequently in the phase II trial, with no significant effect observed, and those with early fibrosis who improved would not be expected to exhibit an improvement in the prediction probability of SomaSignal-determined significant MASH fibrosis (stage F2 or higher). Fibrosis regression is a continuous process; hence it may be speculated that the change in prediction probabilities may be sensitive to changes in fibrosis within stages; further studies including histologic fibrosis using a continuous score and digital pathology are needed to explore this. Moreover, as aforementioned, due to SomaSignal scoring for significant fibrosis, a histologic improvement in fibrosis from stage F3 to F2 would not result in a change in SomaSignal-derived fibrosis, despite this change being clinically meaningful and likely to predict a reduction in liver-related events over time. In addition, because these analyses were conducted in populations with obesity and T2D, rather than in individuals with biopsy-confirmed MASH, the external validity of generalizing these results to the broader MASH population may be limited. This limitation is further compounded by the likely small number of participants with significant fibrosis. Moreover, there may be other patient characteristics that influence the accuracy of the SomaSignal test. It was also beyond the scope of the current analysis to evaluate the performance of the SomaSignal test using classical analytical criteria such as reliability, validity, sensitivity and specificity. A final limitation to note is that, as this was an exploratory *post hoc* analysis, the studies were not primarily designed and powered to assess the outcomes specified.

In conclusion, SomaSignal-derived steatosis was highly prevalent in individuals with overweight/obesity with and without T2D. Semaglutide had a significant treatment effect on SomaSignal-derived MASH components and MASLD stage *vs.* placebo. SomaSignal-derived MASH components were correlated with biopsy-based histologic change, simple liver-related biomarkers and measures of glycemic control, providing supportive evidence that changes in SomaSignal-derived prediction probability may reflect histologic changes. However, further research is required. SomaSignal-derived MASH components may have potential for the non-invasive assessment of treatment effects in early clinical development of MASH therapeutics, or to explore possible effects on MASH-related components in trials where participants have suspected MASH but have not undergone liver biopsy; however, the test is not available clinically and therefore its applicability in clinical practice for diagnostic or patient management purposes remains to be fully evaluated.

## Abbreviations

ALT, alanine aminotransferase; AST, aspartate aminotransferase; eGFR, estimated glomerular filtration rate; FIB-4, Fibrosis-4 index; HbA1c, glycated hemoglobin; HOMA-IR, homeostatic model assessment of insulin resistance; MASH, metabolic dysfunction-associated steatohepatitis; MASLD, metabolic dysfunction-associated steatotic liver disease; OR, odds ratio; PP, prediction probability; RI, renal impairment; T2D, type 2 diabetes.

## Financial support

These trials were sponsored by Novo Nordisk A/S. The sponsor was responsible for the study design; the collection, analysis and interpretation of data and in the writing of the report. The current manuscript is the independent work of the authors and was not commercially influenced by SomaLogic Inc., who as per terms of agreement with Novo Nordisk, provided a medical accuracy review of the manuscript prior to submission.

## Authors’ contributions

MTL, SBN and SL were involved in the concept and design of the article. SL was responsible for the statistical analyses. All authors were involved in interpreting the results of the article, writing and critically reviewing the article. All authors approved of the article and agreed to submit for publication.

## Conflict of interest

JMS declares consultant honoraria from 89bio, Alentis, Alexion, Altimmune, AstraZeneca, Bionorica, Boehringer Ingelheim, Gilead Sciences, GSK, Inventiva Pharma, Ipsen, Lilly, Madrigal Pharmaceuticals, MSD, Northsea Therapeutics, Novartis, Novo Nordisk, Pfizer, Roche, Sanofi, and Siemens Healthineers; speaker honoraria from AbbVie, Academic Medical Education (AME), Boehringer Ingelheim, Echosens, Forum für Medizinische Fortbildung (FOMF), Gilead Sciences, Madrigal Pharmaceuticals MedicalTribune, MedPublico GmbH, MedScape, and Novo Nordisk; and stockholder options in AGED Diagnostics and Hepta Bio. HG declares research grants from AbbVie, ADS AIPHIA Development Services AG Intercept, ARLA Food for Health, and Intercept; declares consulting fees from Ipsen, NOVO, and Pfizer; is a lecturer for AstraZeneca and Eisai; and is on the Data Monitoring Committee of CAMURUS AB. IK, SL, MTL, and SBN are employees and stakeholders of Novo Nordisk A/S. AJS has stockholder options in Durect, Exhalenz, Genfit, Inversago, Rivus, and Tiziana; has served as a consultant to 89bio, Akero, Aligos, Alnylam, Altimmune, AstraZeneca, Boehringer Ingelheim, Eli Lilly, Gilead, Hanmi, Histoindex, Intercept, Madrigal Pharmaceuticals, Merck, Myovant, Northsea, Novo Nordisk, Path AI, Pfizer, Poxel, Promed, Regeneron, Salix, Surrozen, Takeda, and Zydus; his institution has received grants from Boehringer Ingelheim, Echosens, Eli Lilly, Gilead, Hanmi, Intercept, Madrigal Pharmaceuticals, Merck, Novo Nordisk, Salix, and Takeda; and he receives royalties from Elsevier and Wolter Kluwers. MJD has acted as consultant, advisory board member, and speaker for Boehringer Ingelheim, Eli Lilly, Novo Nordisk, and Sanofi; an advisory board member and speaker for AstraZeneca; an advisory board member for Carmot, Pfizer, Roche, ShouTi Pharma, and Zealand; and a speaker for Amgen and Sanofi. MJD has received grants as an investigator in support of investigator-initiated trials from AstraZeneca, Boehringer Ingelheim, Eli Lilly, Janssen, Novo Nordisk, and Sanofi-Aventis.

Please refer to the accompanying ICMJE disclosure forms for further details.
