# Supplementary material for: Proteomic signatures reflect effects of semaglutide treatment for MASH
Source: JHEP Rep. 2025 Jul 22;7(10):101521. doi: 10.1016/j.jhepr.2025.101521 (PMC12447996; doi:10.1016/j.jhepr.2025.101521)
Supplement: Multimedia component 1 [file mmc1.pdf]

Proteomic signatures reflect effects of semaglutide treatment for MASH☆

Jörn M. Schattenberg, Henning Grønbæk, Iris Kliers, Steen Ladelund, Michelle T. Long, Sune Boris Nygård, Arun J. Sanyal, Melanie J. Davies

Table of contents

Table S1 2

Fig. S1 3

Fig. S2 4

Fig. S3 5

Fig. S4 6

Fig. S5 7

Fig. S6 8

Fig. S7 9

Table S1. Baseline characteristics for the full analysis sets.

	STEP 1<br>N = 1,961	STEP 2<br>N = 1,210	Phase 2b trial<br>N = 320
**Age, years**			
Mean (SD)	46.5 (12.5)	55.0 (11.0)	55.0 (10.5)
18 to <65	1,805 (92.0)	953 (78.8)	262 (81.9)
≥65	156 (8.0)	257 (21.2)	58 (18.1)
**Sex**			
Women	1,453 (74.1)	616 (50.9)	194 (60.6)
**BMI, kg/m^2**			
<30	117 (6.0)	211 (17.4)	68 (21.3)
≥30 to <35	643 (32.8)	438 (36.2)	87 (27.2)
≥35 to <40	614 (31.3)	300 (24.8)	165 (51.6)
≥40	587 (29.9	261 (21.6)	165 (51.6)
**eGFR, ml/min/1.73m^2**			
Normal (≥90)	1,332 (67.9)	795 (65.7)	233 (72.8)
Mild RI (≥60 to <90)	601 (30.6)	355 (29.3)	80 (25.0)
Moderate RI (≥30 to <60)	28 (1.4)	59 (4.9)	7 (2.2)
**Race**			
White	1,472 (75.1)	751 (62.1)	248 (77.5)
Asian	261 (13.3)	317 (26.2)	48 (15.0)
Black or African American	111 (5.7)	100 (8.3)	2 (0.6)
Other*	117 (5.9)	42 (3.5)	22 (6.9)

Data are n (%).

*Includes American Indian or Alaska Native, Native Hawaiian or other Pacific Islander, other, and not reported.

BMI, body mass index; eGFR, estimated glomerular filtration rate; RI, renal impairment.

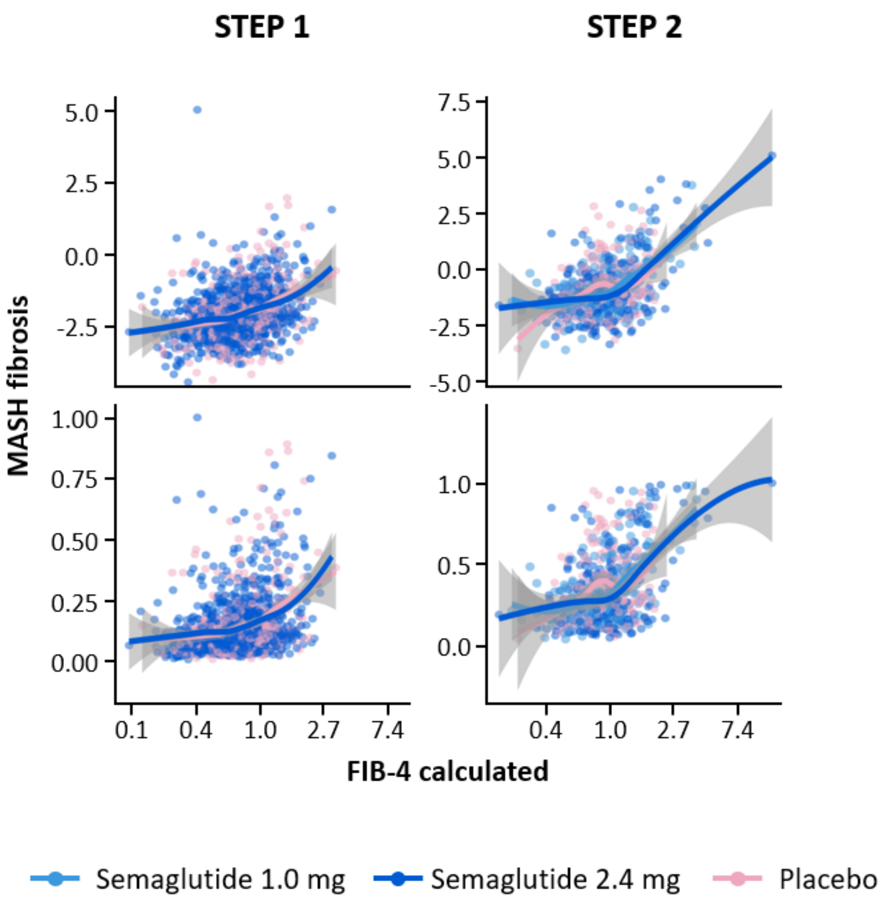

Fig. S1. Correlation between FIB-4 and MASH fibrosis SomaSignal prediction probabilities at baseline for STEP 1 and 2.

Prediction probabilities for SomaSignal-derived MASH components at baseline were derived using the SomaSignal models, with higher probabilities indicating a higher likelihood of more advanced liver pathology. Dichotomized SomaSignal MASH fibrosis: stage 0 to 1 versus 2 to 4. SomaSignal scores provided a probability of whether fibrosis would be greater than or equal to stage 2 (at least clinically significant fibrosis).

FIB-4, Fibrosis-4 index; MASH, metabolic dysfunction-associated steatohepatitis.

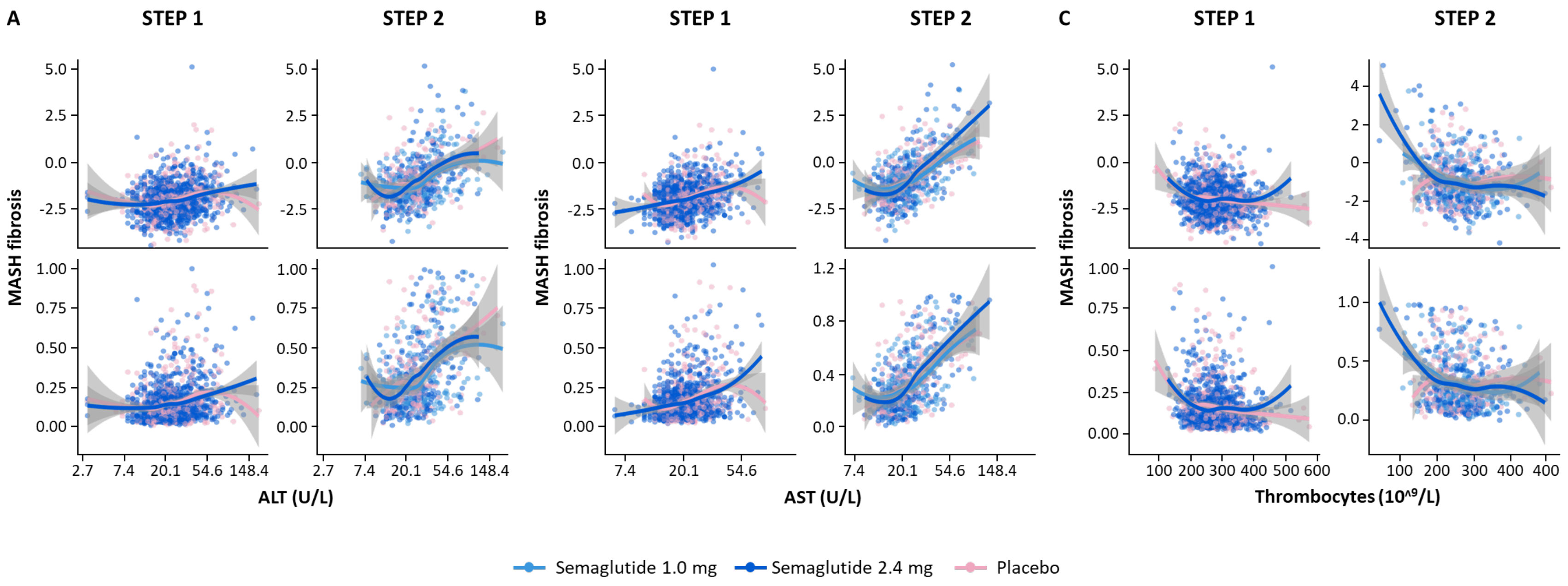

Fig. S2. Correlation between ALT (A), AST (B), and thrombocytes (C) and MASH fibrosis SomaSignal prediction probabilities at baseline for STEP 1 and 2.

ALT, alanine aminotransferase; AST, aspartate aminotransferase; MASH, metabolic dysfunction-associated steatohepatitis.

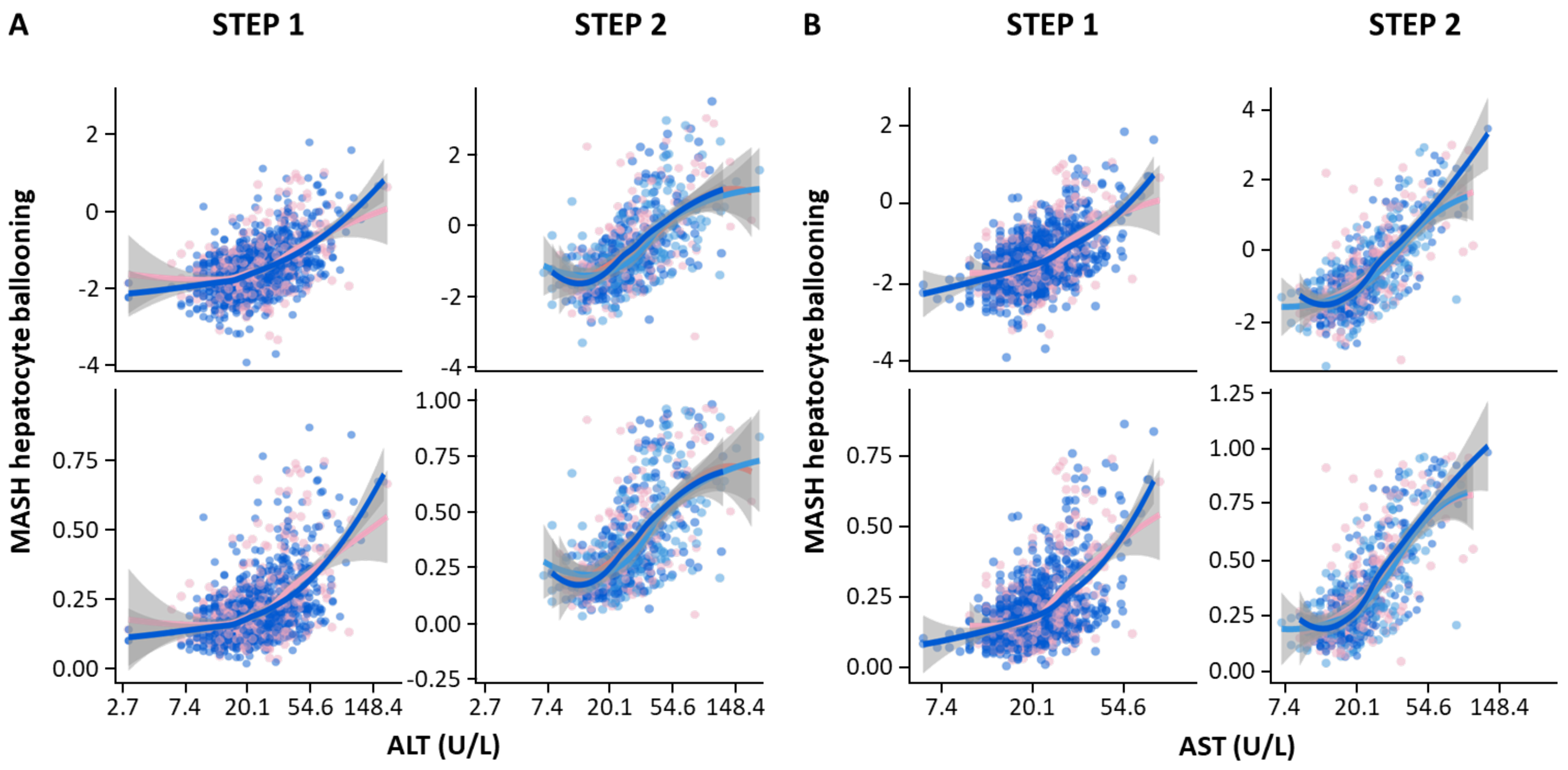

Fig. S3. Correlation between ALT (A) and AST (B) and MASH hepatocyte ballooning SomaSignal prediction probabilities at baseline for STEP 1 and 2.

ALT, alanine aminotransferase; AST, aspartate aminotransferase; MASH, metabolic dysfunction-associated steatohepatitis.

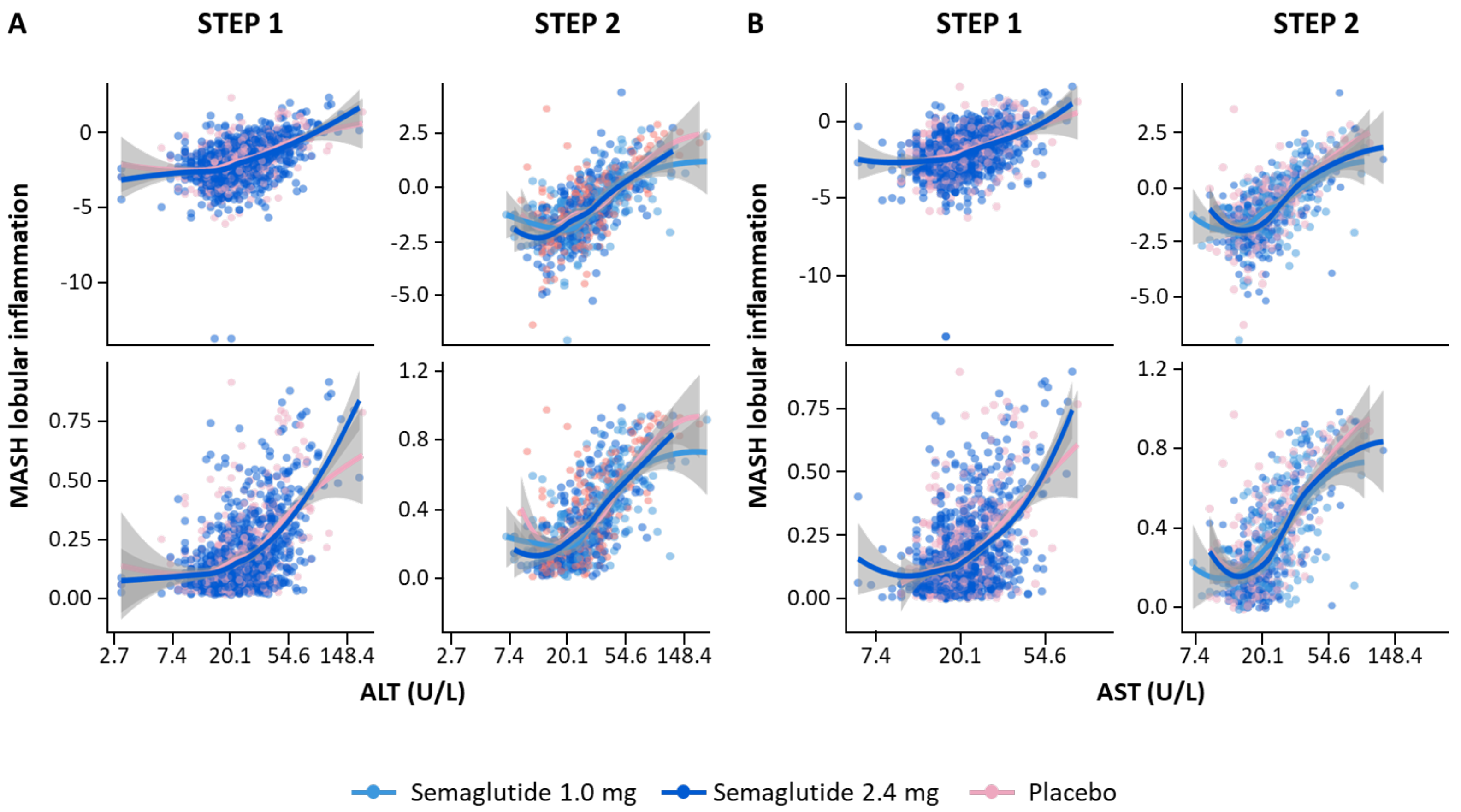

Fig. S4. Correlation between ALT (A) and AST (B) and MASH lobular inflammation SomaSignal prediction probabilities at baseline for STEP 1 and 2.

ALT, alanine aminotransferase; AST, aspartate aminotransferase; MASH, metabolic dysfunction-associated steatohepatitis.

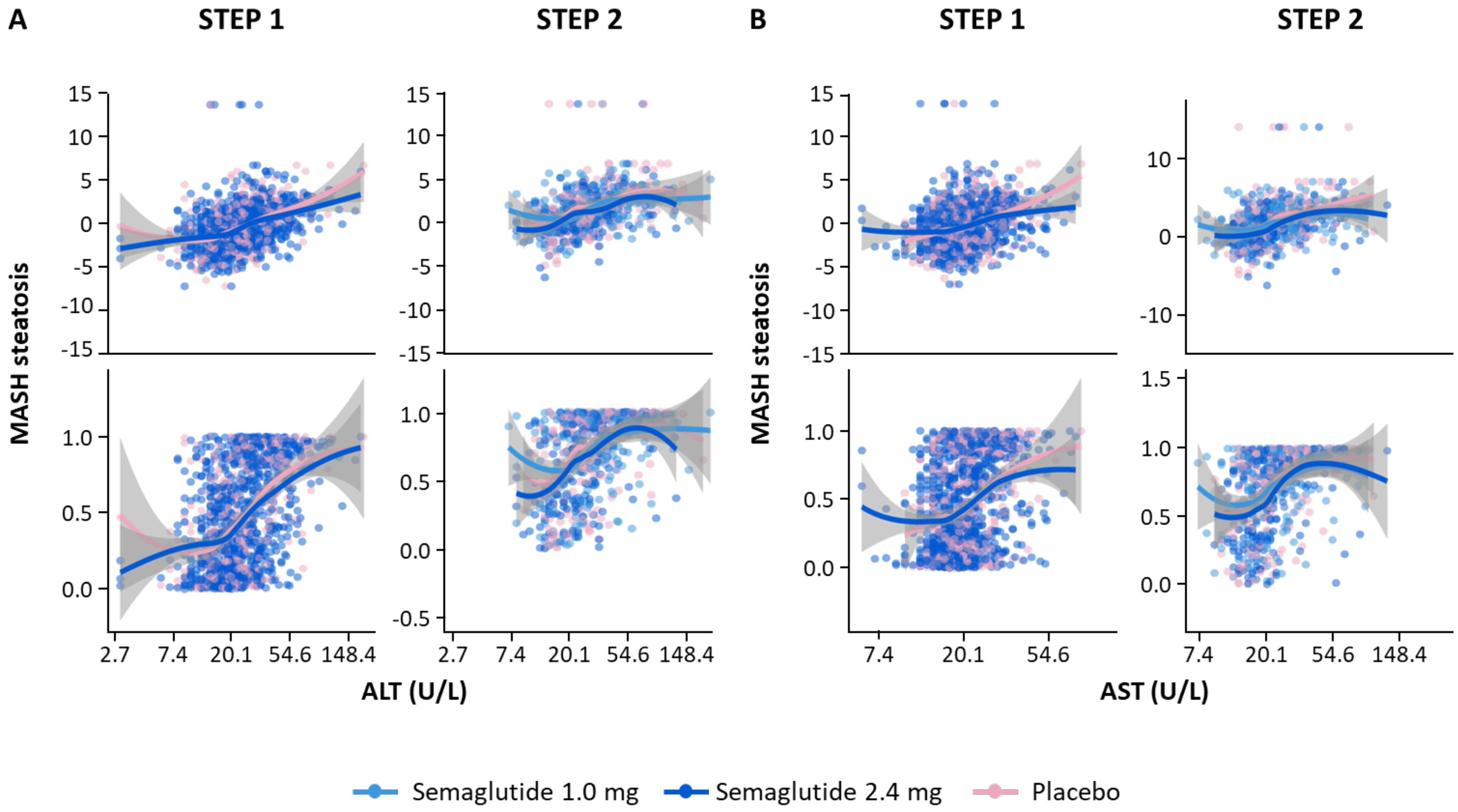

Fig. S5. Correlation between ALT (A) and AST (B) and MASH steatosis SomaSignal prediction probabilities at baseline for STEP 1 and 2.

ALT, alanine aminotransferase; AST, aspartate aminotransferase; MASH, metabolic dysfunction-associated steatohepatitis.

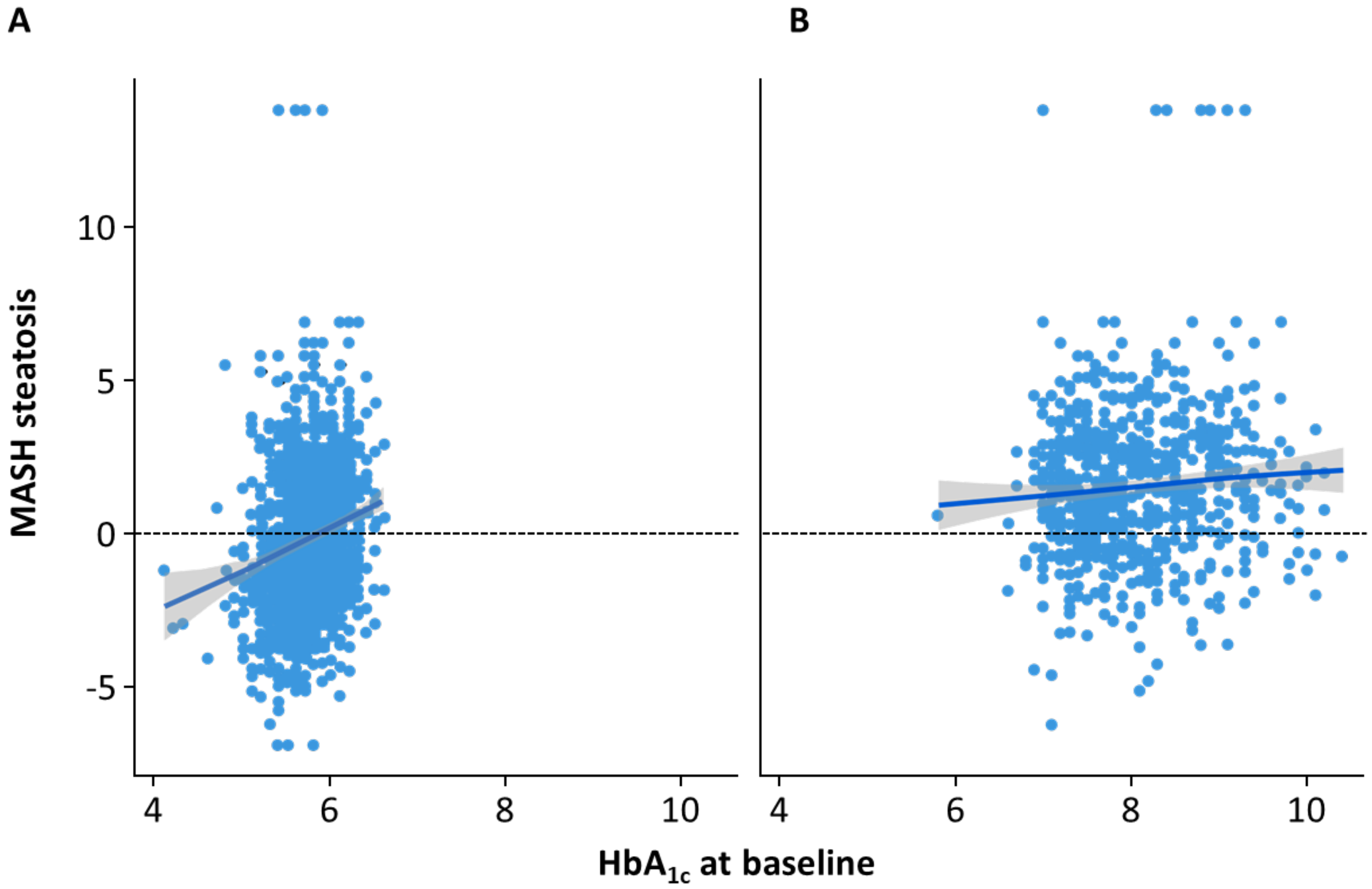

Fig. S6. HbA_{1c} and steatosis SomaSignal prediction probabilities at baseline in STEP 1 (A) and 2 (B).

Prediction probabilities were ≥0.5 (dashed line) based on HbA_{1c}.

HbA_{1c}, glycated hemoglobin; MASH, metabolic dysfunction-associated steatohepatitis.

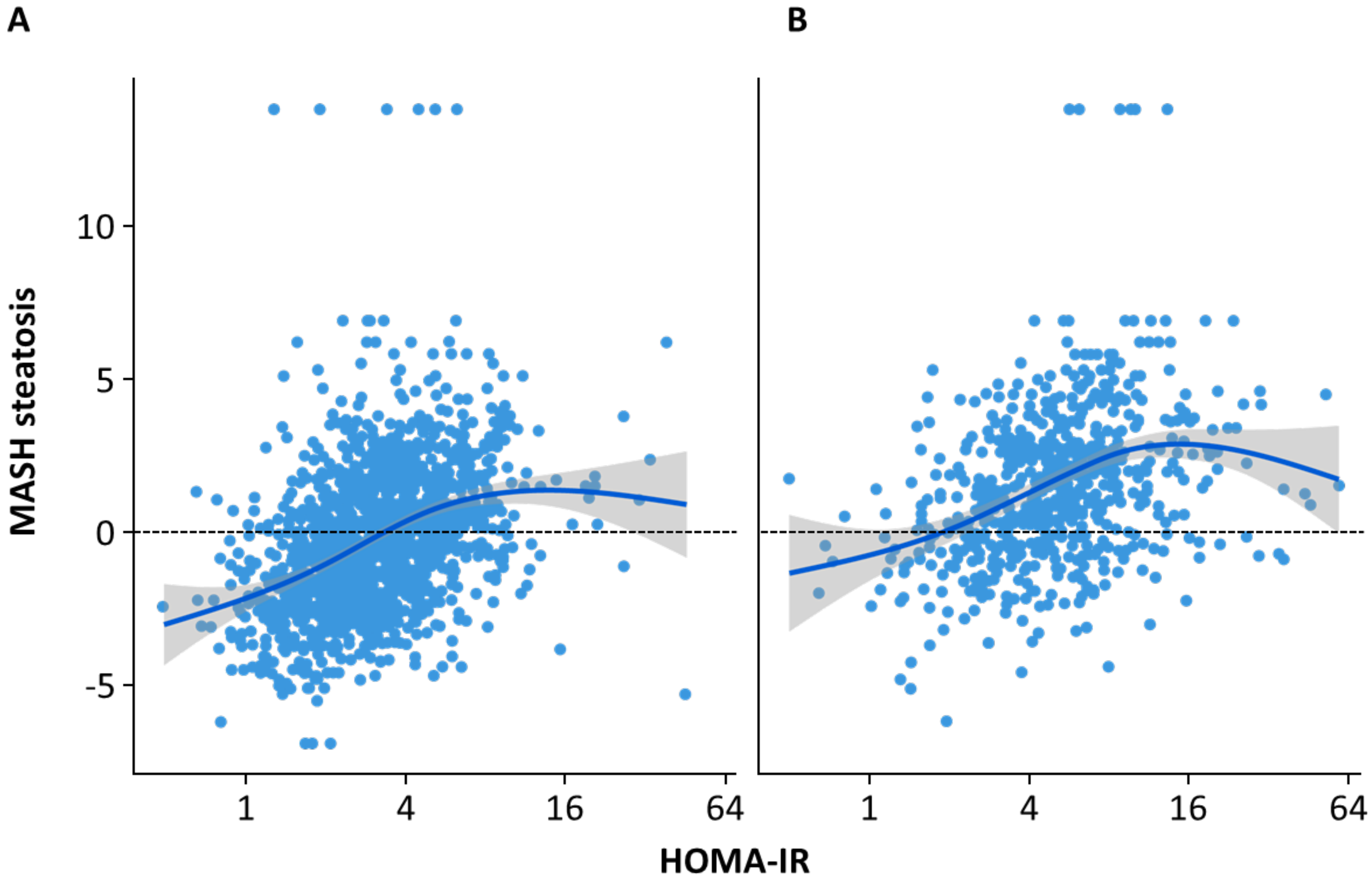

Fig. S7. HOMA-IR and steatosis SomaSignal prediction probabilities at baseline in STEP 1 (A) and 2 (B).

Prediction probabilities were ≥0.5 (dashed line) based on HOMA-IR.

HOMA-IR, homeostatic model assessment of insulin resistance; MASH, metabolic dysfunction-associated steatohepatitis.
